# Flavonoids as Potential Antiviral Agents for Porcine Viruses

**DOI:** 10.3390/pharmaceutics14091793

**Published:** 2022-08-26

**Authors:** Xinwei Zhang, Si Chen, Xue Li, Liying Zhang, Linzhu Ren

**Affiliations:** College of Animal Sciences, Key Lab for Zoonoses Research, Ministry of Education, Jilin University, 5333 Xi’an Road, Changchun 130062, China

**Keywords:** flavonoids, antiviral, porcine viruses, infectious diseases

## Abstract

Flavonoids are types of natural substances with phenolic structures isolated from a variety of plants. Flavonoids have antioxidant, anti-inflammatory, anticancer, and antiviral activities. Although most of the research or applications of flavonoids are focused on human diseases, flavonoids also show potential applicability against porcine virus infection. This review focuses on the recent progress in antiviral mechanisms of potential flavonoids against the most common porcine viruses. The mechanism discussed in this paper may provide a theoretical basis for drug screening and application of natural flavonoid compounds and flavonoid-containing herbs to control porcine virus infection and guide the research and development of pig feed additives.

## 1. Introduction

Swine pathogens, including viruses, bacteria, parasites, mycoplasma, etc., seriously endanger the development of the pig industry. Swine viruses, such as transmissible gastroenteritis virus (TGEV), porcine epidemic diarrhea virus (PEDV), Influenza A virus (IAV), African swine fever virus (ASFV), porcine reproductive and respiratory syndrome virus (PRRSV), porcine circovirus (PCV), and pseudorabies virus (PRV), cause severe diseases through single infection and/or coinfection [1,2,3,4,5]. Furthermore, viruses, such as PCV and PRRSV, are usually the primary pathogens that break through the host’s defense, which generally leads to immunosuppression and causes secondary infection of other pig pathogens [6,7,8]. Therefore, prevention and treatment of virus diseases are regarded as the primary task of the pig industry.

Although vaccination is the primary strategy to prevent some virus infections, vaccination is not feasible or practical in many cases because of virus mutation, diverse virus subtypes, and poor cross-protection effect of vaccines. Furthermore, the antiviral activity of chemical drugs currently used in the clinic is narrow, usually accompanied by cytotoxicity, biological toxicity to organisms, and high cost.

Traditional Chinese medicine (TCM) is widely used in China and Chinese communities outside China, which can effectively relieve the disease from severe to moderate or mild, improve the cure rate, reduce the mortality rate, and promote the recovery of patients [9,10,11,12,13,14,15]. One of the necessary treatments in TCM is taking TCM formula, which is mainly composed of herb medicine (root, stem, leaf, and fruit), animal medicine (internal organs, skin, bones, organs, *etc*.), and mineral medicine. Evidence proved that many herbal medicines have antiviral and anti-inflammatory effects because they contain phytochemicals, such as flavonoids [10,11,12,13]. Excitingly, many studies have confirmed that TCM based on flavonoids and their derivatives have antiviral effects, which can be used in diseases caused by viruses and other pathogens [11,12].

**Figure 1:**
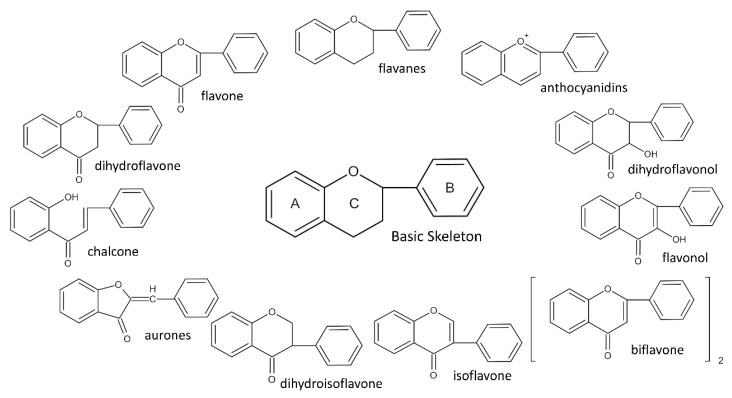
Chemical structure of flavonoids. Flavonoids have three benzene rings, in which A ring and B ring are connected by a three-carbon heterocyclic pyran ring (C ring) to form a basic C6–C3–C6 carbon skeleton. Based on the degree of oxidation and unsaturation of the C ring, flavonoids are divided into flavone, flavanes, anthocyanidins, dihydroflavonol, flavonol, biflavone, isoflavone, dihydroisoflavone, aurones, chalcone, and dihydroflavone, etc.

To date, more than 6000 flavonoid compounds have been identified. Flavonoids have three benzene rings, in which A ring and B ring are connected by a three-carbon heterocyclic pyran ring (C ring) to form a basic C6–C3–C6 carbon skeleton [16,17]. Based on the degree of oxidation and unsaturation of the C ring, flavonoids are divided into flavone, flavanes, anthocyanidins, dihydroflavonol, flavonol, biflavone, isoflavone, dihydroisoflavone, aurones, chalcone, and dihydroflavone, etc. (Figure 1) [16,17]. As phytochemicals, flavonoids have shown potential in chemotherapy because of their special biochemical activities and numerous subclasses, such as anticancer, antioxidant, anti-inflammatory, and estrogen-like effects [11,18,19,20]. In addition, in some cases, flavonoids can directly target virions against the virus infection [11,18,20]. This paper reviewed flavonoids’ antiviral function and mechanism in the porcine virus. The mechanism discussed in this paper can provide a theoretical basis for drug screening and application of traditional Chinese medicine or natural herbs and guide the research and development of feed additives.

## 2. Flavonoids and Their Antiviral Mechanism

Previous studies demonstrated that flavonoids could directly inhibit virus infection via several mechanisms, including interfering with and blocking the processes of attachment, entry, replication, and release [12,20]. Moreover, flavonoids can also evoke the host immune response, regulate the inflammatory response, and block the combination of receptor and virus, thus reducing virus load [11,12,19]. In the following sections, we mainly discussed the recent research progress of flavonoids in inhibiting porcine virus infection (Table 1).

### 2.1. Coronaviruses 

#### 2.1.1. Transmissible Gastroenteritis Virus (TGEV)

TGEV is an enteropathogenic coronavirus that belongs to the *Alphacoronavirus* genus of the family *Coronaviridae* [61,62]. TGEV was first discovered in 1946 in the USA and then spread worldwide, becoming one of the top ten piglet pathogens [61,62,63]. TGEV invades the intestinal epithelium through the mouth, nose, and mucosa of pigs, leading to transmissible gastroenteritis (TGE), characterized by acute intestinal disease with high morbidity and mortality in suckling piglets [61,62]. Therefore, TGE is listed as a reported disease by the World Organization for Animal Health (WOAH). Unfortunately, there is no effective chemical drug to fight against TGEV currently [61,62,63]. In contrast, several studies showed that flavonoids could inhibit TGEV infection [43,64], which provides a good choice for disease prevention and control. 

(+)-Catechin flavonoids, abundantly found in green tea, are bioflavonoids with great potential for anti-oxidative effects. Evidence showed that (+)-Catechin flavonoids inhibit cancer, cardiovascular disease, and virus infection [65]. Liang et al. evaluated the inhibitory effect of (+)-Catechin on TGEV infection in swine testis (ST) cells and found that the viability of ST cells in the TGEV-infected group was markedly increased after adding (+)-Catechin [43]. When the concentration reached 80 μM, the cell survival rate reached a maximum of about 95%. On the contrary, the virus yields in cells treated with (+)-Catechin significantly decreased by almost three log10 units compared with the mock-treated cells. Furthermore, the titer of TGEV in the supernatant of cells treated with an 80 μM concentration of (+)-Catechin was reduced by approximately 237-fold (10^6^ to 10^3.9^). Moreover, intracellular reactive oxygen species (ROS), which are considered to be factors inducing inflammatory responses, were almost entirely inhibited by (+)-Catechin with a concentration of 80 μM [43]. These results indicated that (+)-Catechin could alleviate TGEV-induced ROS and cytopathic effect (CPE) in ST cells. 

In addition, receptor tyrosine kinases (RTKs) play essential roles in cell proliferation [64], and it was verified that (+)-Catechins could target RTKs in cancer treatment [64]. Furthermore, it was reported that the RTK signal pathway was activated during the TGEV infection [44]. These results demonstrate that (+)-Catechin showed significant inhibitory activity on the replication of TGEV in vitro via antioxidation function and the RTK pathway inhibition. Therefore, the antiviral activities of (+)-Catechins against TGEV infection are multiple. It is worth noting that the ST cells are permissive cells for TGEV infection but not the primary target cells. Therefore, whether (+)-Catechin can effectively improve the resistance of intestinal cells to TGEV infection needs to be clarified. 

#### 2.1.2. Porcine Epidemic Diarrhea Virus

Porcine epidemic diarrhea virus (PEDV) also belongs to the *Alphacoronavirus* genus of the family *Coronaviridae,* another top ten swine pathogen, which poses a significant threat to the swine industry in the US and worldwide [61,62,66]. The virus was first identified in 1978 in the UK and spread to most swine-producing areas within a year [61,62,66]. The main transmission route of PEDV is the fecal–oral (direct contact) and/or aerosol (indirect contact) routes, which causes acute vomiting, malabsorption, diarrhea, dehydration, and up to 100% mortality in suckling newborns [66]. Furthermore, due to the emergence of natural recombinant or mutated PEDV [63,67], the efficiency of the PEDV vaccine is controversial. Therefore, an effective agent against PEDV and its emerging variants is urgently needed. 

Quercetin, a phytochemical abundant in fruits and vegetables (especially in onions), has a promising therapeutic prospect. Quercetin has many properties such as antiviral, antioxidative, anti-inflammatory, and anticancer [68]. Li et al. evaluated the effect of quercetin concentrations on the propagation of PEDV strain YN144 and DR13 in the CCL-81 (Vero) cells [31]. First, cells were pretreated with quercetin, followed by PEDV infection, and then the total RNAs of the PEDV were separated and evaluated by qPCR. They found that the amount of viral mRNA decreased dose-dependent on the quercetin concentration. In contrast, when cells were infected with PEDV before quercetin treatment, the viral loads gradually elevated, suggesting that the inhibitory activity of quercetin may exert by interfering with the early events of PEDV replication. Further studies showed that quercetin could suppress the activity of PEDV 3C-like protease (3CL^pro^) through binding with Cys144, Asn141, and His162 residues of PEDV 3CL^pro^ [31]. Moreover, Choi et al. found that quercetin 7-rhamnoside (Q7R), a derivative flavonoid of quercetin, affects the initial stage of PEDV infection [34]. Additionally, several flavonoids, such as flavonoids, dihydromyricetin, isodihydromyricetin, myricetin, herbacetin, rhoifolin, pectolunarin, and ampelopsis grossedentata, can block the enzyme activity of SARS-CoV-2 or SARS-CoV 3CL^pro^ [32,33]. These results indicate that phytochemicals such as quercetin and its derivatives might have potential anti-COVID-19 functions. 

Hyperoside can be extracted from various plants, such as *Hypericum monogynum* and *Crataegus pinnatifida*. Hyperoside is a flavonoid glycoside with multiple pharmacological effects [69]. Recently, Su et al. found that the interaction between PEDV nucleocapsid (N) protein and cellular p53 in the nucleus caused up-regulation of p53 expression, which then activates the p53-DREAM pathway and induces cell cycle arrest at the S-phase, thus resulting in the enhancement of PEDV replication [39]. On the other hand, hyperoside can inhibit PEDV replication and antagonize cell cycle arrest by targeting the viral N protein and interfering with the interaction [39]. These results indicate that small molecular flavonoids targeting viral proteins can effectively resist coronavirus infection. Therefore, it is feasible to screen small molecular flavonoids targeting viral proteins to inhibit virus infection using modern molecular pharmacology technology. However, small molecular agents, such as hyperoside, mainly target one stage of the virus infection or one viral protein, which may limit its inhibitory effect on virus infection. In contrast, the combination of multiple targeted drugs or flavonoids targeting different steps or viral proteins may be more effective in controlling virus infection. 

Puerarin, a medicinal and edible isoflavonoid compound naturally existing in the pueraria, was validated as a medical component with immense therapeutic activities in various health disorders [70]. As reported, 7-day-old piglets were orally administered with puerarin (0.5 mg/kg body weight) on days 5 and 9, followed by orally inoculating PEDV on day 9 [59]. The results showed that puerarin decreased morbidity of piglets infected with PEDV at 3 days post-infection (dpi). Furthermore, oral administration of puerarin can improve the intestinal function of piglets infected with PEDV by increasing the number of total eubacteria, including *Enterococcus* genus, *Lactobacillus* genus, and *Enterobacteriaceae* family, in the intestine but decreasing the number of *Clostridium coccoides* in the caecum [59]. Moreover, oral administration of puerarin also promoted the anti-inflammatory and anti-oxidative activities of the infected piglets [59,60]. Therefore, a dietary supplement of puerarin or Pueraria (Chinese herb Gegen) can effectively prevent and control PEDV infection. 

### 2.2. Influenza A Virus

The influenza virus belongs to the *Orthomyxoviridae* family and includes four genera, *Alphainfluenzavirus*, *Betainfluenzavirus*, *Gammainfluenzavirus*, and *Deltainfluenzavirus*, corresponding to influenza virus A (IAV), B (IBV), C (ICV), and D (IDV), respectively [71]. IAV has a broad host spectrum and is one of the most critical causative pathogens of viral respiratory diseases in avians, humans, and pigs [72,73,74]. In addition, it can spill over from avians to humans and pigs, causing rapid spread and evolution of the virus between and within species [74,75,76]. The surface antigen haemagglutinin (HA) is subdivided into 18 HA subtypes (H1–H18) [73]. The surface antigen neuraminidase (NA) is divided into 11 NA subtypes (N1–N11) [73]. To date, four IAV subtypes, H1 (H1N1, H1N2), H3 (H3N2), H5 (H5N1, H5N2, H5N6), and H9 (H9N2), are circulating worldwide, resulting in up to 100% morbidity within a herd [75,77]. Furthermore, other subtypes, such as H4N1, H4N8, H6N6, H7N2, H7N9, H10N5, and H11N6, were also detected in China or Korea [77]. Although vaccination is one of the effective measures to control swine IAV infection, the antigenic drift of *HA* and *NA* genes of epidemic strains may affect the vaccine’s efficacy [75,77].

Chalcones are α, β-unsaturated ketones which widely exist in plants and have been used in clinical treatment for a long history as bioprecursors of flavonoids. Chalcones presented various pharmacological activities, such as antimicrobial, antiviral, anti-inflammatory, immunosuppressive, etc. [78]. The previous reports show that prenylated A ring and B ring on chalcone flavonoids could inhibit NA activities [51], and non-prenylated chalcone and chalcone-derived flavonoids also showed strong inhibitory activities on H1N1 NAs [49]. Furthermore, chalcone flavonoids target crucial targets of different virus replication processes, such as enzymes in many physiological activities and receptors in pathways activated by virus infection [53,79,80,81,82,83,84,85]. Dao et al. found that chalcones flavonoids, including licochalcone G, licochalcone A, echinatin, 5-prenylbutein, licochalcone D, isoliquiritigenin, licoagrochalcone A, and kanzonol C, extracted from *Glycyrrhiza inflata* showed inhibitory effects on NA from various IAV strains [49]. Among these chalcones, compounds echinantin and isoliquiritigenin showed stronger inhibitory on IAV infection than the other prenylated flavonoids [49]. Furthermore, a synergistic anti-influenza effect was observed when echinatin and oseltamivir were used simultaneously [49]. These results suggest that chalcones flavonoids are NA-targeted inhibitors, interacting with different acting sites of viral NA from oseltamivir.

In addition, epigallocatechin gallate, also called epigallocatechin-3-gallate (EGCG), mainly extracted from green tea, is a polyphenolic flavonoid with the catalysis of epigallocatechin and gallic acid. It was identified that EGCG possesses varieties of biological activities, including antioxidant, anti-inflammatory, and antimicrobial properties [86,87]. Xu et al. found that EGCG could alleviate pathological lung changes, reduce lung wet/dry (W/D) weight ratio, decrease inflammatory cytokine levels, and inhibit myeloperoxidase (MPO) activity in H9N2-infected mice, thus prolonging the survival of mice [28]. EGCG also significantly down-regulated the signaling of toll-like receptor 4 (TLR4) through the laminin receptor [28]. Similar to EGCG, kaempferol is another powerful antioxidant widely distributed in plants, which exerts anti-inflammatory and antioxidation properties by maintaining the activities of various antioxidant enzymes and scavenging free radicals [88,89,90,91]. Therefore, many studies give evidence that TCM-containing kaempferol flavonoids could be potentially applied in clinics to treat chronic inflammatory diseases such as cardiovascular disease, obesity, and diabetes [89,90,91,92]. For example, during H9N2 infection, kaempferol alleviated pulmonary edema, lung wet/dry (W/D) weight ratio, pulmonary capillary permeability, myeloperoxidase (MPO) activity, and the numbers of inflammatory cells in the virus mice model [38]. Furthermore, kaempferol can reduce the levels of ROS, malondialdehyde (MDA), tumor necrosis factor-alpha (TNF-α), interleukin-1beta (IL-1β), and IL-6 in vivo and in vitro but enhance the activity of superoxide dismutase (SOD) [38]. Furthermore, further studies showed that kaempferol inhibits H9N2-induced inflammatory responses by suppressing nuclear factor-kappa B (NF-κB) and mitogen-activated protein kinases (MAPKs) pathways mediated by TLR4/myeloid differentiation factor 88(MyD88) [38]. Thus, EGCG and kaempferol may be promising agents for the treatment of IAV-induced acute lung injury due to their anti-inflammatory, antioxidation, and immunomodulatory properties.

### 2.3. African Swine Fever Virus

African swine fever virus (ASFV), only one member of the *Asfarviridae* family, is a causative agent of African swine fever (ASF). ASF is a highly contagious viral disease of domestic pigs and wild boars, which causes a devastating infectious disease with acute onset, rapid progression, and up to 100% mortality [93,94]. WOAH lists ASF as one of the reported diseases due to its significant impact on the pig industry and the world economy. Notably, ASFV can invade monocytes/macrophages and dendritic cells, thus inhibiting interferon (IFN) expression, regulating cytokine expression and inflammatory response, and thereby avoiding host immune response [94,95]. Unfortunately, to date, there are no effective vaccines or antiviral medicines to prevent and control the disease. 

Myricetin (3, 5, 7, 3′, 4′, 5′-hexahydroxyflavone) is a natural flavonol compound in numerous plants, including oranges, grapes, herbs, and teas, etc. [96]. It has anti-tumor, anti-inflammatory, and antioxidation biological activities [96]. Jo et al. found that flavonols have potential anti-ASFV protease activity [45]. Among them, the most promising flavonol was myricetin, which can effectively inhibit the proteolytic activity of ASFV protease on the viral polyproteins, with a half-maximal inhibitory concentration (IC_50_) of 8.4 μM [45]. The inhibition mostly depends on the 3,4,5-trihydroxyphenyl group of myricetin [45]. Moreover, myricitrin, a derivative of myricetin containing a rhamnoside group, showed a better inhibitory effect on ASFV protease than myricetin, and its IC50 was 2.7 μM [45]. These results indicate that flavonol scaffolds, such as myricetin and myricitrin, can be used as the basic skeleton for developing anti-ASFV agents. 

Apigenin, an abundant fruit and vegetable flavone, belongs to the *Apium* genus. Genkwanin is the O-methyl derivative of apigenin. These flavonoids have various biological activities, such as the induction of autophagy and apoptosis and the suppression of cellular proliferation and inflammatory reactions [97,98,99]. Hakobyan et al. evaluated the antiviral effect of five flavonoids (apigenin, catechin, genistein, luteolin, and quercetin) on ASFV in vitro [47]. The results demonstrated that apigenin had a dose-dependent inhibitory effect on ASFV infection, especially in the early infection stages except in the viral entry process. Furthermore, apigenin decreased the ASFV yield by more than 99.99% when added 1-hour post-infection (hpi) with a concentration of 50 μM. Moreover, apigenin inhibits ASFV-specific protein synthesis and viral factory formation [47]. Based on this finding, several natural apigenin derivatives, including acacetin, apigetrin, genkwanin, rhoifolin, vitexin, and vitexin 2-O-rhamnoside, were screened [48]. As expected, genkwanin can effectively inhibit ASFV infection at the levels of viral early and late proteins and viral DNA synthesis in a dose-dependent manner [48]. Furthermore, genkwanin also suppresses tubulin polymerization to interfere with ASFV transportation along microtubules [48]. These results suggest that genkwanin can inhibit the entry and release of ASFV. Moreover, apigenin can induce ROS production, G2/M cell cycle arrest, and autophagic cell death [98,99], which may enhance the autophagy caused by ASFV proteins, such as E199L, K205R, A137R [10,100,101], resulting in a decrease in ASFV-infected cells and ASFV progeny.

Genistein and kaempferol are the major flavonoids of sophora fruit extracts. Genistein, a phytoestrogen extracted from soybeans, has multimodal action against diseases, including cell cycle arrest, autophagy and apoptosis induction, metastasis inhibition, antioxidant, and anti-inflammatory [102]. Arabyan et al. found that genistein, as a poison of ASFV type II topoisomerase (ASFV-topo II), disrupted viral DNA replication, blocked the transcription and translation of late viral genes, and thus reduced viral progeny [46]. Furthermore, the inhibition was significant in the middle phase of infection (8 hpi) [46]. Further evaluation of molecular docking showed that genistein could interact with four residues (Asn144, Val146, Gly147, and Leu148) of the highly conserved ATP-binding site of ASFV type II topoisomerase, leading to DNA double-stranded breaks and ceasing the viral DNA replication [46]. Meanwhile, kaempferol was evaluated due to its potential inhibitory effect on ASFV out of 90 flavonoids [37]. The results showed that kaempferol could inhibit ASFV infection in the entry and post-entry stages, primarily due to the autophagy induced by kaempferol [37].

### 2.4. Porcine Reproductive and Respiratory Syndrome Virus

Porcine reproductive and respiratory syndrome virus (PRRSV) is an enveloped virus belonging to the *Arteriviridae* family, containing a single positive-stranded RNA of about 15 kb in length [50,103]. PRRSV is the causative agent of the porcine reproductive and respiratory syndrome (PRRS), characterized by reproductive failure, pneumonia, and immunosuppression [50,103]. The disease is one of the most economically significant swine diseases worldwide and is also listed as one of the reported diseases by WOAH [50,103].

Ge et al. found that EGCG, a polyphenolic compound from green tea, exerts multiple roles in suppressing PRRSV infection in a time and dose-dependent manner [22]. First, EGCG can interact with PRRSV to block virus binding to susceptible cells and down-regulate receptors and/or related host proteins to stop virus binding [22]. Second, EGCG also down-regulates pro-inflammatory cytokines to block virus infection in post-infection treatment [22]. Moreover, EGCG inhibits PRRSV proliferation via disrupting lipid metabolism and autophagy [23]. Moreover, EGCG palmitate showed a higher inhibitory effect on PRRSV infection than EGCG and ribavirin in both pre-treatment and post-treatment [24]. These results indicate that EGCG is effective against PRRSV infection pre-, post-, or co-treatment. 

Ruansit et al. reported that quercetin modulated the immune responses by enhancing type I and II interferon and type I interferon-regulated genes (IRGs) and decreasing pro- and anti-inflammatory cytokine expressions against highly pathogenic (HP)-PRRSV challenge in vivo and in vitro [29]. In addition, piglets immunized with the PRRSV-1 modified-live virus (MLV) vaccine followed by oral administration of quercetin exhibited a significant reduction of HP-PRRSV viremia compared with the untreated group [29]. Furthermore, Heat shock protein 70 (HSP70) is essential for PRRSV infection [30]. However, quercetin can inhibit the expression of HSP70 [30]. These findings suggest that as an effective oral immune-modulator, quercetin may assist the PRRSV-1 MLV vaccine in alleviating clinical symptoms induced by HP-PRRSV and enhancing immune defense against HP-PRRSV in the host [29,30].

Rutin is a quercetin glycoside in several medicinal and eatable plants, which can effectively improve metabolic function due to its effective antioxidation and anti-inflammatory properties [104]. Rutin can reduce TNF-α, IL-6, cyclooxygenase-2, IL-1β, and other pro-inflammatory markers, block the activation of NF-κB/MAPK pathways, effectively relieve inflammation and improve metabolic function [104]. Suebsaard et al. evaluated the immunomodulatory effect of rutin on monocyte-derived macrophages infected with HP-PRRSV [21]. As expected, HP-PRRSV inhibits the expressions of immune-related genes, including *myxovirus resistance 1*, *interferon regulatory factor 3* (*IRF3*), *IRF7*, *2′-5′-oligoadenylate synthetase 1*, *stimulator of interferon genes* (*STING*), *osteopontin* (*OPN*), *IFNα*, *IFNβ*, *IFNγ*, and *TNFα* [21]. However, the expression of *IRF3*, *IRF7*, *STING*, *OPN*, *IFNα*, *IFNβ*, and *IFNγ* genes significantly increased, and the levels of *TNFα* and *TGFβ* were reduced in the HP-PRRSV-infected group after being stimulated with rutin [21]. These results demonstrated that rutin inhibits PRRSV infection via enhancing IFN responses and reducing pro- and/or anti-inflammatory reactions, indicating an immunomodulatory role of rutin during PRRSV infection.

Isobavachalcone (IBC) is a flavonoid extracted from the plant *Psoralea corylifolia*, which was used in TCM for several centuries [52,53,105,106]. As a phytochemical flavonoid with various biological activities, IBC was used in many diseases such as acute myeloid leukemia, colitis, and osteoporosis. A recent report showed that IBC could induce apoptosis and inhibit cell proliferation by suppressing the protein kinase B (AKT)/Glycogen Synthase Kinase-3 (GSK3)β/β-catenin pathway in cancer cells [105]. These demonstrate that IBC might have a variety of activities, including anticancer, anti-inflammatory, antioxidative, antibacterial, antifungal, and antiviral activities [106]. Wang et al. found that IBC can inhibit PRRSV infection at the post-entry stage by suppressing the initiation of viral RNA replication but did not interfere with the viral attachment and entry [52]. However, the exact mechanism needs to be elucidated in the following studies. 

Xanthohumol (Xn) is a prenylated flavonoid isolated from hops *Humulus lupulus* L., which has broad biological activities, including anti-inflammatory, inhibition of cholesterol accumulation, and inhibition of cell proliferation [54]. Recently, Liu et al. found that Xn can play an anti-PRRSV role by inhibiting virus adsorption and internalization [54]. Moreover, Xn can up-regulate the genes related to the antioxidant reaction, including *nuclear factor-erythroid 2-related factor 2 (Nrf2), heme oxygenase 1* (*HMOX1)*, *glutamate-cysteine ligase catalytic subunit (GCLM)*, and *NAD(P)H quinone oxidoreductase 1 (NQO1)* [54,55]. After that, the Nrf2-HMOX1 signaling pathway was further activated to alleviate the viral-induced oxidative stress [54,55]. In addition, Xn can down-regulate the expression of IL-1β, IL-6, IL-8, and TNF-α in PRRSV-infected cells and can also effectively relieve clinical symptoms, lung pathology, and inflammatory reactions in lung tissues of piglets induced by HP-PRRSV infection [54,55]. The results indicate that Xn may be a therapeutic agent against PRRSV infection.

Soy isoflavones (ISF), including genistein and daidzein isoflavones, have many potential pharmacological activities. For example, ISF suppresses arterial stiffness, regulating intestinal flora and mimicking estrogen via its special structure to affect the physiological state of animals [107,108]. In addition, recent studies showed that ISF demonstrated immune modulation, anti-inflammatory, and antiviral properties [56,57,58,109,110]. Rochell et al. evaluated the effects of dietary soybean meal (SBM) on the growth performance and immune response of pigs infected with PRRSV [57]. The results showed that pigs fed high concentrations of SBM during PRRSV infection had improved average daily growth (ADG), more elevated hematocrit and hemoglobin concentration, but lower viremia, haptoglobin, and TNF-α in serum [57]. Furthermore, Smith et al. found that ISF supplementation may enhance the ratio of cytotoxic-to-helper T-cell, increase PRRSV-induced neutrophilia, and elicit neutralizing responses, thus activating adaptive immune responses and reducing the mortality of PRRSV-infected pigs [56,58]. These results indicate that dietary ISF is beneficial to the recovery and elimination of PRRSV infection. Therefore, dietary supplementation of soy-derived isoflavones is a potential health feed additive for the pig industry.

### 2.5. Porcine Pseudorabies Virus

Porcine pseudorabies virus (PRV) belongs to the *Varicelloviru* genus, the *Alphaherpesvirinae* subfamily, and the *Herpesviridae* family [111]. It has a broad spectrum of hosts, including most mammals and avians, causing Aujeszky’s disease, which is characterized as fatal and central nervous system disorders, respiratory symptoms of fattening pigs, abortions of pregnant sows, and fetal death [111,112]. In addition, PRV can infect humans and cause severe clinical symptoms, such as acute human endophthalmitis and encephalitis [113,114,115]. Moreover, due to the emergence of high pathogenic PRV variants, the available vaccines cannot provide adequate protection for swine against PRV infection [25]. Currently, there are no antiviral drugs to treat PRV infections.

Several groups reported that flavonoids, including quercetin, kaempferol, EGCG, dihydromyricetin (DMY), and luteolin, have anti-PRV activities via various mechanisms [25,26,35,36,116,117]. Sun et al. found that quercetin can significantly inhibit a broad spectrum of PRV isolates in a dose-dependent manner via interaction with the viral envelope glycoprotein D (gD protein), which engages in the recognization of host receptors [35]. Quercetin injection protected mice from the lethal challenge and reduced the viral load and mortality of PRV-infected mice [35]. Furthermore, quercetin significantly reduced the secretion of ROS induced by PRV [36]. The miRNAs induced by PRV (such as SSC-mir-450c-3p and novel-m0400-3p) regulated the decrease in ROS, especially thioredoxin interacting protein (TXNIP) and nitric oxide synthase 2 (NOS2) [36]. The reduction of ROS, especially TXNIP and NOS2, was regulated by miRNA (ssc-miR-450c-3p and novel-m0400-3p) induced by quercetin [36]. Moreover, Li et al. reported that kaempferol inhibited PRV replication in the mouse brain, lung, kidney, heart, and spleen by inhibiting the transcription of immediate early gene *IE180* and the expression of latency-associated transcripts, thus alleviating the pathological changes in these organs [25]. In addition, kaempferol can induce the serum levels of IL-1β, IL-4, IL-6, TNF-α, and IFN-γ to increase, reaching the peak on the third day and decreasing to the normal level on the fifth day [25]. Huan et al. demonstrated that EGCG could inhibit PRV infection in vitro and in vivo in a dose-dependent manner [26]. Further studies showed that 50 μM EGCG can efficiently block PRV adsorption, entry, and replication of PRV in cells, and 40 mg/kg EGCG had a 100% protective effect on mice infected with PRV before or after treatment [26]. Moreover, isobavachalcone can suppress PRV infection by blocking cell-to-cell fusion in the late stage of PRV infection [53]. 

Dihydromyricetin (DMY) is a flavonoid extracted from several plants, such as *Ampelopsis grossedentata* (*A. grossedentata*) and *Nekemias grossedentata* (*N. grossedentata*) [118]. DMY has pharmacological effects such as anti-inflammation (NLRP-3, NF-κB, cytokines, and neuroinflammation), antioxidation, improving mitochondrial dysfunction, and autophagy regulation [118]. DMY can be metabolized into three metabolites by the gut microbiota, which then modulates gut microbiota composition [119]. Sun et al. found that DMY inhibits PRV infection by blocking viral entry and suppressing pyroptosis by PRV induced [116].

Luteolin is a natural dietary flavonoid found in *Verbascum lychnitis*, *Carex fraseriana*, and other herbs, exhibiting antioxidant, anti-inflammatory, apoptosis-inducing, chemopreventive, and antiviral activities [117,120,121]. Furthermore, Liu et al. found that luteolin can inhibit the inflammatory response in PRV-infected cells [117]. Further studies showed that luteolin could inhibit the activation of STAT1/3-dependent NF-κB and induce the expression of Nrf2-mediated HO-1 [117]. In addition, Luteolin also inhibits the production of pro-inflammatory mediators nitric oxide (NO) and inflammatory cytokines and the expression of their regulatory genes, such as *nitric oxide synthase (iNOS) and cyclooxygenase-2 (COX-2)* [117]. Therefore, flavonoids can be used to control PRV infection, including quercetin, kaempferol, EGCG, dihydromyricetin, and luteolin. Further studies should focus on the effect of these flavonoids on PRV infection in piglets.

Meanwhile, widely used herbs in TCM, such as *Polygonum hydropiper* L., *Garcinia species*, and *Licorice*, also exhibit anti-PRV activity due to the abundant antiviral ingredients in these herbs [40,122,123]. For example, several flavonoids, including rutin, quercetin, hyperoside, quercitrin, galloyl quercitrin, quercitrin, kaempferol, anthraquinones, naphthoquinones, and sesquiterpenoids, were identified in the ethyl acetate fraction of *Polygonum hydropiper* L. (FEA) [40,124]. Further studies showed that FEA could significantly inhibit the synthesis of NO, and down-regulate the expressions of inflammatory factors, such as *iNOS and COX-2*, and cytokines in PRV-infected cells [40]. Moreover, FEA reduced the transfer of NF-κB to the nucleus and the phosphorylation of MAPK [40]. These results suggested that FEA interfered with the inflammatory responses induced by PRV through the NF-κB/MAPK signaling pathway. 

Additionally, Adnan et al. extracted bioactive compounds from *Garcinia parvifolia* leaf using ethyl acetate (45 L Ea), ethanol (45 L Et), and hexane (45 L H) solvents, respectively [123]. As a result, six phytochemical ingredients, including saponin, flavonoid, tannin, phenolic, terpenoid, and steroid, were confirmed in 45 L Et extract. In addition, five components, including flavonoid, tannin, phenolic, terpenoid, and steroid, were identified in 45 L Ea extract, whereas only two compounds, terpenoid, and steroid, were found in 45 L H extract. The evaluation of these three extracts showed that the Ea extract exhibited the highest antiviral activity (75%) at 125 μg/mL, followed by the Et extract (26%) [123]. In contrast, the H extract has the lowest antiviral activity against PRV and the highest cytotoxicity [123]. Furthermore, both ethyl acetate and ethanol extracts can inhibit viral attachment and completely inactivate PRV [123]. These results also indicate that ethyl acetate is the best solvent for extracting antiviral compounds from herbs.

### 2.6. Porcine Circovirus 2

Porcine circovirus 2 (PCV2) is a non-enveloped DNA virus with a diameter of about 20 nm, belonging to the genus *Circovirus* in the family *Circoviridae* [5]. PCV2 is an etiologic agent of porcine circovirus disease (PCVD) and porcine circovirus-associated disease (PCVAD), which further be characterized as a post-weaning multi-systemic wasting syndrome (PMWS), porcine respiratory disease complex (PRDC), porcine dermatitis and nephropathy syndrome (PDNS), enteric disease, and reproductive disease [5,6]. In addition, PCV2 is an immunosuppressive pathogen, which can lead to secondary infection of other pathogens in pigs, including swine viruses, bacteria, and mycoplasma [5,6]. To date, nine subtypes of PCV2 have been identified, including PCV2a to 2i [6,125]. Although the PCV2 vaccine is effective to some extent and the cross-protection between different PCV2 subtypes was reported [126], the virus is still prevalent in swine farms worldwide [127,128]. 

*Spatholobus suberectus Dunn* (*S. suberectus*) is a widely used herb in traditional Chinese medicine, which can improve blood circulation and be anti-platelet, anti-inflammatory, antibacterial, neuroprotective, and anticancer [41]. Chen et al. treated PCV2-infected cells with total flavonoids of *S. suberectus Dunn* (TFSD) extracted from *S. suberectus* [41]. They found that the increase in oxidative stress molecules (NO, ROS, GSSG) and oxidative stress enzymes (SOD and MPO) induced by PCV2 infection decreased to normal levels in the TFSD-treated group in vitro [41]. In contrast, the decrease in GSH and SOD caused by PCV2 infection recovered to normal levels in the TFSD-treated group [41]. Furthermore, the immunomodulatory and antioxidant effects of TFSD were further confirmed in PCV2-infected mice [42]. These results indicate that TFSD, an antioxidant and antiviral agent, can provide immune protection during PCV2 infection.

EGCG also exhibits anti-PCV2 activity [27]. Li et al. found that EGCG directly targeted PCV2 virions with an affinity constant of about *Kd* = 98.03 ± 4.76 μM, blocking the binding of virions with heparan sulfate, a cellular receptor on the surface of host cells [27]. Furthermore, the results of molecular docking showed that the critical amino acids of the viral capsid could form the binding pocket for EGCG reorganization and binding, among which four residues, including ARG51, ASP70, ARG73, and ASP78, especially two arginines, were crucial for the binding of EGCG and viral capsid [27]. These results indicate that EGCG is effective in the early stage of PCV2 infection and can be used for prevention or treatment in the early stage of the infection. Moreover, EGCG is also one of the antioxidation and immune-regulation agents [129]. Therefore, the antioxidation activity of EGCG might also be worked against PCV2 infection.

## 3. Limitations of Flavonoids as Antivirals in Pigs

Although flavonoids have exhibited effective inhibition of virus infection, some limitations must be overcome. 

First, most flavonoids are extracted from plants containing various bioactive ingredients in the extract. However, although advanced technologies, such as microwaves, ultrasound, pressurized liquids, supercritical fluids, and electric fields, are used to extract flavonoids, different bioactive components could be obtained by disparate extraction techniques or the same techniques under other conditions [123,130]. For example, bioactive compounds from *Garcinia parvifolia* leaf using ethyl acetate (45 L Ea), ethanol (45 L Et), and hexane (45 L H) solvents are distinct and showed diverse antiviral activities on PRV infection [123]. Therefore, extraction methods and quality control of flavonoids are crucial.

Second, the effective concentration of each flavonoid is critical for its antiviral activities. Some natural flavonoids may be metabolized into another bioactive ingredient in vivo, the fundamental antiviral components. However, characteristics of natural flavonoids such as poor water solubility, high instability, and low oral bioavailability limit their application. Therefore, new delivery strategies, such as nanospheres, nano-capsules, micro and nano-emulsions, micelles, solid lipid nanoparticles, and capsules [12], are promising, and it is necessary to evaluate the economy/cost and applicability of these delivery strategies in pigs. Farmers most favor a low-cost and effective method.

Notably, whether flavonoids have cytopathic effects in vitro or adverse effects in vivo is controversial. Some groups reported that when flavonoids were used in non-cytotoxic concentrations in vitro or in vivo, flavonoids could inhibit virus infection in a dose-dependent manner, thus improving the survival rate of cells or animals or providing preventive protection against virus attack [25,35,37,53,59]. However, others believe that the use of flavonoids or prescriptions should be restricted because the antiviral mechanism of flavonoids, especially flavonoid-containing plants, is still unclear, and the side effects are unknown. Therefore, it is urgent to identify safe and environmentally friendly flavonoids and natural compounds or herbs against the porcine virus. 

## 4. Conclusion and Perspectives

**Figure 2:**
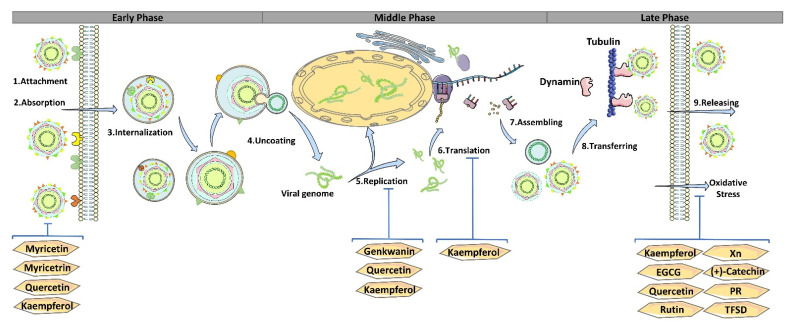
Antiviral activities of flavonoids on swine virus infection. Different flavonoids play antiviral roles at the various stages of virus infection by interacting with viral protein directly or eliciting host immune responses and inflammatory reactions. In addition, several flavonoids, including kaempferol, quercetin, and EGCG, have broad-spectrum antiviral functions.

To date, more than 30 kinds of swine viruses, including 20 emerging or re-emerging swine viruses, have been confirmed to infect pigs and cause severe diseases, seriously affecting the swine industry and the world economy. Unfortunately, there are no effective drugs and treatments for most of these viruses, and some existing prevention programs, including vaccines, have certain limitations or low effectiveness. This paper reviewed the antiviral activities of flavonoids against porcine viruses in vivo and/or in vitro. Generally, flavonoids inhibit porcine virus infection by inhibiting viral attachment, entry, replication and translation, assembly, and/or release (Figure 2). Meanwhile, flavonoids can trigger antioxidation and modulate immune responses and inflammatory reactions against virus infection. However, the detailed molecular mechanism of flavonoids against swine viruses also needs to be clarified using molecular docking and molecular dynamics (MD) simulation.

Some flavonoids play antiviral roles in multiple stages of the virus infection and can be used to prevent and treat the diseases. Flavonoids, such as EGCG, kaempferol, rutin, IBC, and quercetin, exhibit antiviral activities against numerous porcine viruses, indicating that these flavonoids have broad-spectrum antiviral activities. Multiple targeted flavonoids mainly modulate cellular inflammatory, oxidation, and immune responses. In contrast, some are single target agents, such as hyperoside and chalcones, which are only effective in a single step of virus infection and only act on a single viral protein. Notably, the same flavonoid may inhibit different viruses in different ways. For example, quercetin inhibits PRRSV infection by immunomodulatory, anti-inflammatory, and antioxidant activity but inhibits PEDV infection by direct interaction with PEDV protein. However, most published data on flavonoid antiviral activities are based on in vitro studies. Therefore, future research should focus on field applications of flavonoids and their derivatives against porcine viruses, including efficacy, safety, dosage, and application stages. 

Moreover, the combination of flavonoids extracted from herbs and berries showed synergistic antiviral effects, further confirming the effectiveness of flavonoids containing-TCM in the antiviral activities against swine viruses. Therefore, oral administration of purified active ingredients of flavonoids or dietary supplementation of the plant containing flavonoids is promising for preventing and treating virus infection in the swine industry.

## Figures and Tables

**Table 1:** Antiviral activities of flavonoids against swine viruses.

Flavonoid	Structure	Virus	Treatment Time	Experimental Model	Effective Concentration	Inhibitory Phase	Mechanism	Reference
epigallocatechin-3-gallate (EGCG)	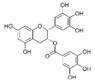	PRRSV	Pre	In vitro	125 μM	Early phase	Block PRRSV binding to cells, reduce pro-inflammatory factors, and disturb lipid metabolism	[21,22,23,24]
		PRV	Pre	In vivo/in vitro	50 μM (in vitro) 40 mg/kg (in vivo)	Multiple steps	Inhibit PRV adsorption, entry, and replication	[25,26]
		PCV2	Pre	In silico/in vitro	100 μM	Early phase	Interacts with heparan sulfate to competitively inhibit capsid binding	[27]
		H9N2	Post	In vivo	10 mg/kg	Multiple steps	Reduce Organs damage, inflammation, and virus titer	[28]
quercetin	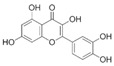	PRRSV	Post	In vivo	10 mg/kg 100 μM in vivo	Multiple steps	Cross protective efficacy and inhibit the activity of Hsp70	[29,30]
		PEDV	Pre	In vitro	100 μM	Early phase	Inhibit the activity of PEDV 3CL protease	[31,32,33,34]
		PRV	Pre Simultaneity	In vitro In vivo	50 μM (in vitro) 1.51 μg (in vivo)	Early phase	Interacting with the viral gD protein. Reducing the secretion of reactive oxygen species induced by PRV.	[35,36]
kaempferol	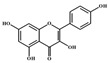	ASFV	Simultaneity Post	In vitro	20 μg/mL	Early phase	Induced autophagy	[37]
		PRV		In vitro	240 mg/kg	Early phase	Reduced the expression level of viral IE180 and inhibit viral replication	[25]
		H9N2	Post	In vivo	15 mg/kg	Later phase	Inhibit the NF-κB and MAPKs pathways mediated by TLR4/MyD88 NF-κB	[38]
hyperoside	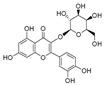	PEDV	Pre	in vitro	20 μM	Later phase	Inhibited N protein-induced S phase cell cycle arrest	[39]
rutin	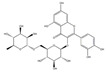	PRRSV	Post	In vitro	7.8 μg/mL	Later phase	Regulate inflammation and suppress PRRSV replication	[21]
		PRV	Post	In vitro	40 μg/mL	Later phase	Inhibit the activation of NF-κB and MAPK pathways	[40]
total flavonoids of S. suberectus Dunn (TFSD)	-	PCV2	Post	In vitro/In vivo	50–100 μg/mL (in vitro)/ 50–100 mg/kg (in vivo)	Later phase	Anti-oxidation and immunomodulatory effects	[41,42]
(+)-catechin	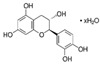	TGEV	Post	In vitro	80 μM	Later phase	Anti-oxidation	[43,44]
myricetin	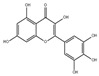	ASFV	Simultaneity	In vitro	20 μM	Early phase	Interaction of 3, 4, 5-trihydroxyphenyl with ASFV protease	[45]
myricitrin	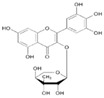	ASFV	Simultaneity	In vitro	40 μM	Later phase	Interaction of 3, 4, 5-trihydroxyphenyl with ASFV protease	[45]
genistein	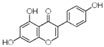	ASFV	Pre	In vitro	50 μM	Early phase	Disrupt the synthesis of viral DNA	[46]
genkwanin	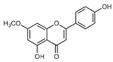	ASFV	Simultaneity	In silico/in vitro	40 μM	Early phase, later phase	Inhibition of ASFV entry and release phases	[47,48]
apigenin	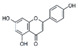	ASFV	Pre	In vitro	50 μM	Early phase, later phase	Expression of 25-kD virus protein was inhibited	[47,48]
quercetin 7-rhamnoside (Q7R)	-	PEDV	Simultaneity	In vitro	10 μg/mL	Early phase	Inhibit the early stage of viral replication	[34]
chalcone	-	H1N1	Pre	In vitro	2.49 ± 0.14 μg/mL	Later phase	Noncompetitive inhibitors of H1N1 neuraminidase	[49,50,51]
Isobavachalcone (IBC)	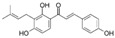	PRRSV	Post	In vitro	<15 μM	Later phase	Interference with RNA synthesis	[52]
		PRV	Simultaneity	In vitro	25.6 μM	Later phase	PRV replication was inhibited at the intercellular fusion stage	[53]
Xanthohumol (Xn)	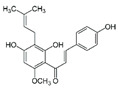	PRRSV	Pre	In vivo/in vitro	15 μM	Later phase	Nrf2-HMOX1 axis	[54,55]
Isoflavones (ISF)		PRRSV	Post	In vivo	1600 mg/kg	Later phase	Supported immune responses	[56,57,58]
Puerarin		PEDV	Post	In vivo	0.5 mg/kg	Later phase	Alleviate systemic inflammation	[59,60]

## Data Availability

All data generated or analyzed during this study are included in this published article.
